# Report of Observations Made in the British Hospitals in Belgium

**Published:** 1817-05

**Authors:** 


### PART II.

### COMPREHENSIVE ANALYTICAL REVIEW OF MEDICAL LITERATURE.

"Tros, tyriusve, nobis nullo discrimine agetur."

### *Report of Observations made in the British Hospitals in Belgium.* By JOHN THOMSON, M. D. &c. &c.

( Continued from page 252. )

1. *Wounds of the Diaphragm.* Wounds of the thorax are often complicated with those of the diaphragm. The latter are certainly not necessarily fatal; since, in several cases, seen in the Belgic Hospitals, the diaphragm must have been perforated once, if not twice, by musquet balls. In one case the ball had entered the right hypochondriac region, under the edge of the false ribs, and came out on the right side of the spine, on a level with the superior edge of the os ilium. This patient spat blood for some days, and voided it also by stool.

On the examination of a patient who died 30 days after receiving the wound, and in whom a ball had entered the chest on the lower and outer part of the right papilla, making its exit from the abdomen on the left side of the umbilicus, the right lobe of the lung was found wounded, and the diaphragm and upper part of the right lobe of the liver perforated. No symptom of *risus sardonicus* appeared in any of these instances of wounded diaphragm.

2. *Wounds of the Parietes of the Abdomen.* There were many examples of the great difficulty of ascertaining whether or not a ball has entered the cavity of the abdomen, owing to the circuitous route which the ball sometimes makes through the parietes; or to its passing through the cavity without materially injuring any of its contents. A man was struck by a splinter of a shell on the right buttock. A hardness and swelling took place over the right hypochondrium, and in the right epigastric region. The tumefaction subsiding, a large body was felt between the extremities of the right false ribs and the navel. It was cut down upon, and a piece of shell, nine ounces in weight, was extracted on the 25th day after the receipt of the wound. It had occasioned but a slight degree of peritoneal inflammation. A ball entered at the anterior extremity of the cartilages of the false ribs on the right side, but had not passed out. After some weeks, a tumour formed on the left side of the back whence the bullet was extracted. Vomiting, and pain in the epigastrium for some days after the injury. The great danger of wounds in the parietes of the abdomen arises from the extension of inflammation to the peritoneum; hence the utility of evacuations and low regimen.

3. *Wounds of the Liver.* Twelve cases of hepatic wounds were convalescing in the Belgic Hospitals. Most of them had two orifices. In a case with only one opening, there was a considerable discharge from the wound of a serous fluid tinged with bile. Bile was spat up. In another, the ball penetrated through the anterior part of the eighth rib on the right side, and was supposed to be lodged in the spleen, from the pain felt in that region. Bile, nearly pure, was discharged from the wound for many days. In consequence of the high inflammatory symptoms, more than 120 ounces of blood were abstracted during the first ten days. On the fifteenth, a hæmorrhage took place from the wound, by which the patient lost 20 ounces. The hæmorrhage recurred to the same extent on the 31st day, with a slight accession of inflammatory symptoms, which were removed by two copious venæsections. Purgatives were constantly required in this case. At the end of the eighth week, the bilious discharge had ceased. In one singular case of wound in the liver, which proved fatal on the 30th day, it was found that the ball had entered the chest below the right papilla—passed through the lower part of the lungs, and upper part of the right lobe of the liver—entered the abdomen, and come out by the side of the umbilicus. The injured lung had collapsed, and was covered with coagulable lymph. A quantity of bile had been accumulated between the liver and peritoneum covering its convex surface, in a circumscribed space resembling an abscess. In several instances, wounds of the liver were complicated with wounds of the lungs, and bile discharged through the thoracic wound.

Jaundice did not attend wounds of the liver; but the skin was sallow in many instances. In a case that terminated fatally on the 20th day, the ball was found lodged in the substance of the liver, without jaundice. Two cases of gun-shot wounds of the spleen terminated fatally, one from hæmorrhage through the external wound — the other into the abdomen.

4. *Wounds of the Stomach and Intestines.* Two recoveries were seen from wounds of the stomach; one, a lance wound; the other by a musquet ball. In both, the contents of the stomach had issued through the wounds for some days after the injury.

In twelve recoveries from wounds of the intestines, no external protrusion took place. In most cases the balls had passed through and out of the abdomen. In two cases they lodged. In one of these the ball entered the left groin; the fæces passed through the wound seven days, then resumed their usual course. In another, the entrance of the ball was just above the root of the penis, through which the fæces were discharged in considerable quantities. In two instances balls were voided by stool. In some of the intestinal wounds the fæces passed by one opening, in others by both. In one case, for instance, a ball entered the left groin, and passed out of the right buttock; the fæces were discharged through both openings. — Wounds of the small intestines are generally fatal, either primarily or secondarily; those of the large intestines often heal easily. But two distinct examples of convalescence from wounds of the small intestines were seen. In one, the ball entered by the right ant. sup. spin. proc. of the ilium, passing out to the left of the umbilicus. A quantity of yellowish bilious-looking fæces was discharged for several weeks by both orifices. The man, though much emaciated, was recovering. In another, who was convalescent, the ball had entered three inches above the posterior spinous process of the ilium, on the left side, and passed out of the right hypochondriac region near its middle. Part of his food came out by the posterior orifice for fifteen days. In a very singular case, where a musquet ball had entered the abdomen below the left side of the umbilicus, and made its exit on the same side, near the spinous process of one of the lumbar vertebræ, a small quantity of fæces continued, when Dr. T. left Belgium, to be discharged by the posterior opening, through the whole depth of the muscles of the loins. Air and urine came also with the fæces. In another wound in the back, in the region of the kidney, urine was discharged by the orifice for twenty-five days.

That in wounds of the intestines, whether from violence or sphacelus, Nature is the best Surgeon, has been proved by a variety of experiments, facts, and reasonings. It cannot, however, be too forcibly urged, that copious blood letting and the antiphlogistic regimen are the best *auxiliaries* which the practitioner can employ in all injuries of the abdominal viscera.

5. *Wounds of the Bladder.* Fourteen recoveries from musquet-ball wounds of the bladder were seen. In most cases the ball had passed through and out of the pelvis. In three cases the balls were lodged in the bladder. In two cases the bladder wounds were complicated with wounds of the intestines. In one of these the ball had entered the tip of the right buttock, and came out of the middle of the left groin. The urine in this patient was discharged partly by the wound in the groin and partly by the rectum. The patient had a healthy appearance, and seemed to experience very little inconvenience from the injury. In most cases of wounded bladder the urine came by both openings. The continued use of the flexible catheter was of great service where the patient would wear one. In one case, where the catheter was neglected, an abscess formed from infiltration of urine into the upper and inner part of the thigh. The abscess when opened, continued to discharge urine. In several cases, the balls appeared to pass through the pelvis without injuring either bladder or intestines, or producing much constitutional or local affection.

6. *Wounds of the Penis and Testes.* In one case a musquet ball had passed from side to side, through the body of the penis. The wound healed kindly. In two cases, a portion of the urethra was carried away by a shot. The wound healed over a catheter. In several instances, balls had passed through one or both testes. One young man, in whom a musquet ball passed through both testes and occasioned great swelling and pain in these organs, was affected with violent hysterical paroxysms.

7. *Wounds of the Loins and Pelvis.* These were very much diversified. In some, the wounds healed readily; in others, abscesses formed in the canals of the shot, and passed to a great distance from the original wounds, requiring various openings. Many cases presented themselves, in which the balls had obviously passed through a greater or less portion of the pelvis. In one case, the ball had entered the right buttock immediately behind the trochanter major, and was found lodged under the integuments in the middle of the left groin, from whence it was afterwards extracted. In two instances the balls had entered on the inside of the anterior superior spinous process of the ilium on the right side, and passed out of the right buttock a little above and behind the trochanter major. In another case, a ball entered the inner part of the right groin, and passed out at the middle of the right buttock. In a fifth, a ball had entered the symphysis pubis, and passed out at the tip of the right buttock. In these, and various other similar instances, the only wonder is how the great vessels should have escaped!

In several of the gun-shot wounds across the loins and through the region of the pelvis, complete or partial paralysis of the lower extremities was produced. In many of the pelvic wounds in which a cure seemed to be taking place, their orifices were filled up with fungous granulations, indicating either the lodgement of some foreign body, or exfoliation of bone. Death had taken place in two instances by secondary hæmorrhage, supposed to proceed from branches of the gluteal arteries.

8. *Wounds of the Hip-joint.* In some wounds in the region of the hip-joint, a discharge of synovia put it beyond all doubt that the capsule had been opened by the balls. In others, it was doubtful. In almost all, great swelling had taken place, frequently extending over the whole limb. In one case, the ball had entered over the right trochanter major, and passed inwards to the joint. The discharge of synovia was, at first, considerable, but gradually diminished, and recovery was taking place. In another case, in which it was doubtful whether the ball had entered the joint, there was considerable swelling in the site of the great trochanter, with but little in the region of the acetabulum. On an incision being made into the swelling, the ball was found embedded in the neck of the femur, from which it was extracted. A large abscess had formed in the posterior part of the thigh. On this patient dying, the neck and head of the thigh bone, together with the acetabulum, were found in a diseased state. In an injury of the hip-joint occasioned by a ball, an exfoliation from the side of the acetabulum had taken place, and the symptoms, both local and constitutional, were so mild as to afford hopes of recovery by anchylosis. Many patients were sinking under hectic fever, in whom extensive suppurations had taken place in the region of the hip-joint previously to death.

9. *Wounds of the Thigh.* In several instances of cannon shot wounds, great swelling and protrusion of the muscles of the thigh took place. Where the wounds were small, as by grape or canister shot, the external protrusion overlapped the edges of the wound, producing, to a certain degree, the strangulation of the muscles. In these cases, the return of the muscles was promoted by the attention to the position of the limb, by gentle pressure, and by moderating the inflammation.

Numerous examples were seen of musqet balls penetrating the thigh in all directions. Most of them healed kindly; in some instances, however, high degree of erythematic inflammation, occasioning great swelling of the thigh, leg, and foot, attended. Secondary hæmorrhage from sloughing of the great vessels was not uncommon; and, at later periods, hospital gangrene. Three cases occurred of wounds of the thigh, complicated with fracture of the bone, in which secondary hæmorrhage proved fatal about the fifth day. Several cases of secondary hæmorrhage, from the 20th to the 30th day, were seen in gun-shot fractures of the thigh. In one case, from the femoral artery so high up as to render it necessary to tie the external iliac. There were several instances where it was necessary to tie the superficial femoral artery. In some of these, gangrene of the foot and leg supervened. Some cases required amputation. The femoral vein, in one instance, was tied in consequence of sloughing from hospital gangrene. Several remarkable instances occurred, where balls ran across and near to the blood-vessels of the thigh, without either primary or secondary hæmorrhage. Dr. T. saw two cases were the femoral vessels were exposed by foreign bodies, without hæmorrhage. A great proportion of the wounds of the thigh were complicated with fracture of the bone. This is very dangerous where the fracture is above the middle of the bone, or near either of the extremities. In those cases where the fractures were above the middle, shortening of the limb, displacement of bones, and distortion took place to a great extent. Counter-openings were very useful in the ulterior stages of these compound fractures of the thigh.

In gun-shot fractures of the thigh, the limb may, in general, thinks our author, be placed in the semi-bent position, without splints or bandages, till the 20th day, or even till a later period. It was only when inflammation had subsided, and the process of re-union was about to take place, that advantage was derived from placing the limb in the extended position, or the application of splints and bandages; and even then, great care and caution are necessary to prevent œdema and gangrene. It is only by placing the limb in the extended position, that it is possible to judge of the degree of shortening and distortion which may have taken place.

Excellent effects were produced in several instances by the application of rollers to the foot and leg, while the soft parts more immediately surrounding the fracture were gently compressed by the eighteen-tailed bandage, applied so as to admit of, and to facilitate the discharge of matter from the abscesses and sinuses formed in the neighbourhood of the fractures. Of the apparatus for permanent extension, Dr. Thompson does not entertain a very high opinion.

"It must be left to future experience to determine whether this apparatus is not a thing better calculated to amuse the rich, than to be of real service to the poor." 135.

Dr. T. lauds the great pains which the French surgeons bestow upon the management of fractured limbs, in respect to daily dressing; to the condition, position, and bandaging of the limb.

10. *Wounds of the Knee-joint.* Dr. T. witnessed more than sixty examples of this kind of wound—mostly inflicted by musket-balls; a few by grape and canister, some by the lance. In a majority of cases, the local and constitutional symptoms were peculiarly severe. Several had died of these wounds, and others were in imminent danger of doing so, before the symptomatic fever could be so far abated as to justify amputation. Great pain, tension, and swelling of the joint itself, were usually accompanied by œdema of the foot and leg; and not unfrequently with erythematous swelling of the whole limb; sometimes extending up to the trunk of the body, rendering amputation impossible. These wounds terminated sometimes in extensive abscesses round the knee, and in the cavity of the ham; sometimes in erysipelas; sometimes in gangrene of the foot and leg. In a few rare instances, balls had passed through, or even lodged in the joint, or in the ends of the bones, without the patients appearing to have suffered much from constitutional fever, or local inflammation. Dissection in several instances, shewed that balls, in passing through the joints, had fractured the ends of the bones, occasioning in the synovial membrane and cartilages, appearances resembling scrofulous affections of these joints. In the progress towards recovery, the wounded knee and other joints acquired in external form and in feel, a resemblance to joints affected with chronic inflammation, or white swelling. How many of the cases in which these changes had taken place would yield to the usual remedies, or ultimately require amputation, it would be curious and useful to ascertain.

11. *Wounds of the Leg.* The local and constitutional symptoms accompanying compound fractures here bore a great resemblance to those of the thigh, except that the symptoms were less severe, and the chance of ultimate recovery greater. Some of the worst cases were, where the fracture was near the ankle joint, to which the inflammation had spread. Most of these fractures were *comminuted;* in few instances did balls pass through the tibia without fracturing it. In one remarkable case, the ball passed through the tibia five inches below the knee, without fracturing the bone. Several balls had lodged in the tibia. A ball in one case had entered the upper, fore, and outer part of the leg, and seemed to have taken a direction backwards to the ham. Various attempts were made to discover it with a probe introduced into the wound, but without success. An abscess, after some time, formed over the inner and upper extremity of the gastrocnemius muscles, from which, upon opening it, a bit of the pantaloons was taken out. Small fungous granulations protruded for some time from the extremities of the wound, but these yielded to the application of escharotics and pressure, and the wounds healed up without the ball being discovered, or any sensible exfoliation of bone having taken place. Whether the ball will ever discover itself is one of those problems which time only can solve. 141. In one case, a ball had entered the head of the right tibia and passed out at the middle and outside of the leg. Secondary hæmorrhage coming on, a longitudinal, and afterwards, a transverse incision was made over the exit of the ball, by which the extremities of the peroneal artery were exposed and tied. Great swelling of the divided muscles took place, but the hæmorrhage did not recur.

12. *Wounds of the Ankle and Foot.* Wounds of the ankle-joint are nearly as severe as those of the knee, and the symptoms and appearances are so similar, that they need not be repeated. The constitutional effects, however, though occasionally severe, were much less so, in general, than in gun-shot wounds of the knee. Though in many cases recovery was advancing, yet it was evident that amputation would be the result in a large proportion. The wounds of the foot present nothing interesting.

13. *Wounds of the Shoulder.* In one of these, the ball had entered the middle of the clavicle, its route or situation being undiscovered, without producing any considerable injury of the chest or shoulder. In another case, an iron canister shot had entered and fractured the right clavicle; passed through, and was cut out from under the spine of the scapula; the wound bled profusely, the patient vomited blood copiously on being wounded, and spat blood for three weeks. He was recovering.

In one very remarkable case, an iron shot, about an inch and a half in diameter, had entered the fore part of the left shoulder, below the clavicle, and about two inches from its outer extremity, and passed out behind at the external edge, and a little below the middle of the scapula. The pulsations of the subclavian artery were at first visible in the wound, but had ceased to be so when we last saw this patient; the ball seemed to have passed on the upper and outer side of the artery. This patient could move his fingers, but had no power over the arm or shoulder.

A variety of cases occurred, in some of which the shoulder-joint had been struck, and others, in which it had been laid open by grape-shot and by cannon-balls. In some of these cases amputation had been performed; in others, attempts were making to cure the injuries without the removal of the limb. In one case, a ball had carried away the integuments on the upper and fore part of the joint, together with the greater part of the deltoid muscle, and had shattered the head of the humerus. The head of the humerus exfoliated, and about seven weeks after the injury, four inches of the upper part of that bone were removed from the socket. Pains were taken to bring the soft parts together by means of adhesive straps. The appearances were favourable beyond what could be expected in an injury so severe.

In one very remarkable case, the ball had entered on the inner side of the insertion of the deltoid, and come out at the root of the neck, immediately above the inner extremity of the clavicle, appearing in its course to have passed through the axilla and under that bone. A large portion of the first rib exfoliated; but neither hæmorrhage, paralysis, nor injury of the parts contained in the chest, seemed to have been produced.

Dr. T. saw various cases in which, in consequence of severe injuries of the shoulder-joint, amputation had been performed on the field during the action; and others, in which it had become necessary to perform that operation at subsequent periods. A few seemed to be in a state of recovery in whom balls had actually passed through the shoulder-joint; and several in a state which would probably require amputation on the subsidence of the secondary constitutional symptoms by which they were attended. Even in the greater part of those cases which had the most promising appearance, a great degree of swelling continued to exist, accompanied by extensive suppurations, fungous protrusions from the wounds, and exfoliations of the bones, with their usual constitutional symptoms.

14. *Wounds of the Arm.* Two instances occurred of secondary hæmorrhage from the brachial artery. In both, it was necessary to tie that vessel. In one, the pulse had returned at the wrist, after several weeks. In the second, sphacelus of the fingers, and gangrenous inflammation of the fore arm were produced, and the arm was amputated above the elbow, but *below* the ligature.

15. *Wounds of the Elbow Joint.* These were very numerous, by sabre, lance, and ball. In almost all, great swelling, pain, and tension of the joint, with œdema of the fore-arm and hand took place. In many cases, from abscesses and exposure of the bones, amputation would be necessary. Those in which the wounds were healed up, or were closing, seemed to be passing into a state resembling that of white swelling; and would probably require amputation at last. In others, the more favourable termination in anchylosis was to be expected. In one case, a ball was *lodged* in the elbow joint, without any great degree of swelling or inflammation. The olecranon, in another case, had been dissected out, and the patient was doing well.

16. *Wounds of the Fore-Arm and Hand.* In several of these, from gun-shot, secondary hæmorrhage had occurred, and required amputation. In wounds of the palm of the hand, collections of matter frequently took place under the palmar aponeurosis, and extended up the fore-arm along the sheaths of the tendons. Many of the wounds of the metacarpal bones healed readily. Those of the bones of the thumb were, like those of the bones of the great toe, accompanied with severe local and constitutional symptoms.

We have thus analysed in the most minute and circumstantial manner, every interesting fact in the practical part of the volume before us. The remaining 120 pages are occupied in the discussion of the question, whether amputation should be performed before or after inflammation? and also, in what cases this operation is necessary?

Dr. T. has given a very able historical sketch of the different opinions which have swayed the chirurgical world from Du Chesne, the first military surgeon who recommends immediate amputation, down to Guthrie. Our author himself is decidedly in favour of the *latter* practice; and although it was not so *comparatively* successful in the Belgic warfare, as in the Peninsular campaigns; yet the protracted pain, suffering, and danger of those in whom amputation became ultimately necessary and practicable, and the far greater proportion even of *these* who died, than of those who had undergone the operation at an early period, were circumstances so evident and striking, as to occasion many regrets among the army surgeons, that primary amputation had not been more frequently performed.

The hurry, confusion, and uncertainty which occur during a battle; the multiplicity and variety of the cases which demand attention, and the shortness of time for deliberation, render it extremely desirable and proper that the military surgeon should be armed with fixed rules, easily remembered, and readily applicable to each case.

The following are those cases which Dr. T. thinks indisputably require immediate amputation.

I. Where a limb has been shot off.

II. Where the bones or joints have been fractured, or much bruised by cannon balls.

III. Where large portions of the soft parts have been removed with the division of the principal blood-vessels and nerves, or with the denudation and contusion of bone.

IV. Where, without the division of the skin, the bones have been fractured by the impulse of a spent ball, and the soft parts severely contused and disorganized.

V. Where the division of the principal artery of a limb, accompanied by fracture of the bone, has been produced by a musquet-bullet; and,

VI. Where musquet-bullets, in passing through the large joints, have not only torn and lacerated the ligaments, but have also fractured the articulating surfaces of the bones.

Speedy death by hæmorrhage, gangrene, or symptomatic fever is the usual consequence of No. 1; and few examples of success in such cases were seen, where early amputation had not been performed. Injuries of the *sixth* kind did not, in general, prove so speedily fatal; but without immediate amputation, a small proportion only will be found to recover. In Belgium, some of those who had suffered *this* injury, died during the first attack of inflammation and fever; some were sinking under hectic and diarrhœa; the remainder passing into a state that would ultimately require amputation.

Besides these six classes, it is evident that there are numerous other wounds, where primary or secondary amputation will be necessary, and where the decision must depend on the judgement of the practitioner; and be influenced by the site of the wound; the number, nature, and extent of the parts injured; the effects which such injuries are likely to produce; and the favourable or unfavourable situation of the patient.

We shall glance at some of the more prominent features of this part of our author's work. Wounds of the wrist joint, where the articulating surfaces, particularly of the radius, are laid bare and fractured; wounds in which musquet-bullets pass through, and lodge in the ankle-joint; fracture of both bones of the forearm, with division of the radial and ulnar arteries; fracture of both bones of the leg by bullets; cases where bullets pass through the ends of the tibia; or have fractured this bone near the knee or ankle-joint; where a bullet is lodged deep in the tibia; fractures of the tibia with wounds of the arteries of the leg; these, in our author's opinion, require amputation in the first instance. This only applies, however, to military surgery; for in private life, or where the sick would be safely lodged in an hospital, some of these injuries might no doubt do well.

Wounds of the elbow-joint, in which the bullets pass through that joint and fracture the articulating surfaces of the bones. Sabre wounds, where the bones of the joint are severely injured; wounds in which bullets have passed through the knee-joint and fractured the articulating surfaces of the bones; where the bullet, in fracturing the humerus, divides the artery, or without dividing the artery, passes through the bone near either of its extremities; where bullets have passed through the cavity of the shoulder joint, and splintered the articulating surfaces of the humerus or scapula. Gun-shot fractures of the thigh, particularly when the fracture has its seat above the middle of the bone; these, Dr. T. thinks, are proper subjects for primary amputation.

We have thus given a most minute detail of Dr. Thomson's work; which, after all, is but a "*Catalogue raisonné*" of the wounds observed in the Belgic hospitals. Many of the outlines noticed in this book, will, in the course of the present year, be amply filled up by a Gentleman of acknowledged abilities and unlimited experience;* a Gentleman who, through many a disastrous campaign and bloody battle, observed with care, and recorded with fidelity, every interesting phænomenon which this wide scene presented to his view. The result of his labours will form the brightest gem in the crown of military surgery.

Dr. Thomson's well-earned reputation will not suffer by the present performance; nor will it be greatly enhanced thereby; unless we except the historical sketch of amputation, which is well drawn up and evinces both erudition and research. The rest is merely a record of facts; a catalogue of human woes—a melancholy memento of our national valour in the BATTLE OF WATERLOO!

---

* Dr. Hennan, Deputy Inspector of Hospitals.

